# Supplementary material for: High-impedance microwave resonators with two-photon nonlinear effects
Source: Nat Commun. 2025 Jan 9;16:552. doi: 10.1038/s41467-025-55860-8 (PMC11718305; doi:10.1038/s41467-025-55860-8)
Supplement: Supplementary file 1 — Supplementary Information [file 41467_2025_55860_MOESM1_ESM.pdf]

Supplementary Information for the Article Titled "High-Impedance Microwave Resonators with Two-Photon Nonlinear Effects"

S. Andersson,[1] H. Havir,[1] A. Ranni,[1] S. Haldar,[1] and V. F. Maisi[1,*]

[1] *NanoLund and Solid State Physics, Lund University, Box 118, 22100 Lund, Sweden*

(Dated: November 27, 2024)

In this supplemental material, we present additional data on the reflected signal to complement the material in the main article. Furthermore, we present the measurement data used for the calibration described in the Methods section in the main article.

SUPPLEMENTARY NOTE 1: REFLECTION MEASUREMENTS

Supplementary Figure 1 shows the measured reflection coefficient R response corresponding to the transmission response of Figs. 2 a-b and d-e in the main article. These measurements were made early on in the experiment with

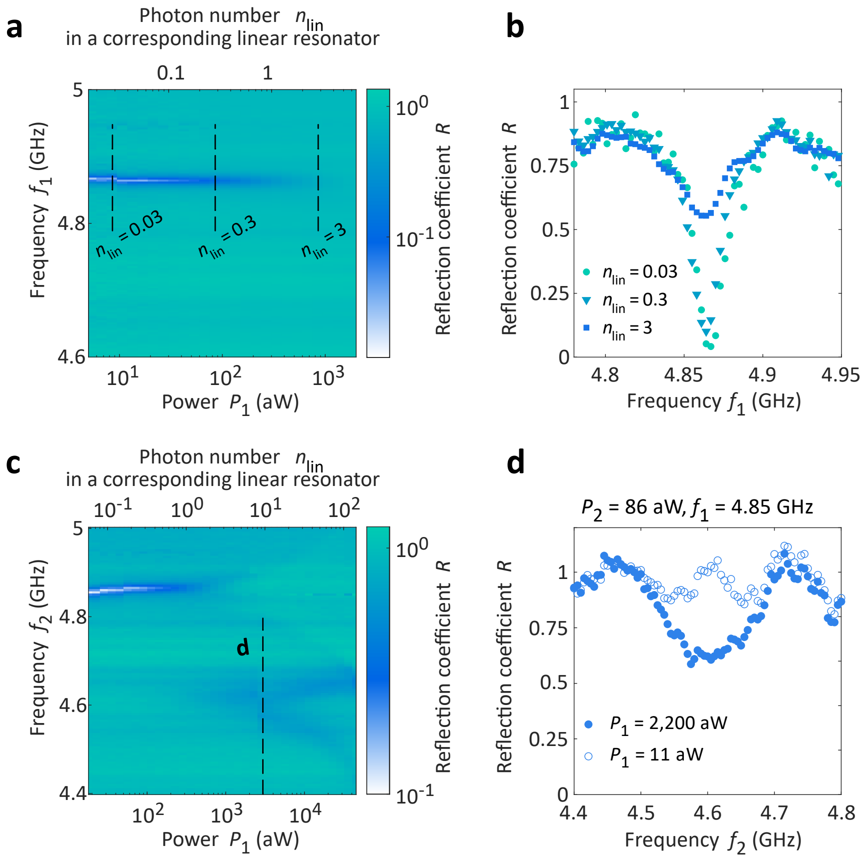

Supplementary Figure 1. **a,** The reflection coefficient R measured as a function of drive frequency f_1 and drive power P_1. The top scale shows the average photon number $n_{\rm lin}$ in a corresponding linear resonator. The white dashed lines indicate the line-cuts shown in Supplementary Figure 1b. **b,** Line-cuts at similar powers as Fig. 2b of the main text. **c,** Two-tone measurement showing the reflection coefficient R of the second RF tone as a function of drive power P_1 of the first tone and frequency f_2 of the second tone. The drive frequency is fixed at $f_1 = 4.85$ GHz and the power of the second tone is fixed at $P_2 = 86$ aW. **d,** Line-cuts of the reflected signal of the second tone at powers $P_1 = 2,200$ aW and $P_1 = 11$ aW.

* ville.maisi@ftf.lth.se

$\Phi = 0.4\Phi_0$ to characterize the device. In Supplementary Figure 1a we see that when the input power P_1 approaches the power at which a fully linear resonator has an average number of one photon ($n_{\text{lin}} = 1$), the reflection coefficient increases as photons are unable to enter the resonator and therefore have to be reflected back. This is also depicted in Supplementary Figure 1b which shows three line-cuts indicated by white dashed lines in Supplementary Figure 1a. Note that these powers are similar to those in Fig. 2b in the main text. In Supplementary Figure 1a some horizontal stripes are visible. These appear due to standing waves arising in the cabling, causing constructive or destructive interference of the signal. Furthermore, at $n_{\text{lin}} = 0.03$ there is a jump in the resonance frequency due to a spurious shift in the magnetic field through the SQUID loop.

Supplementary Figure 1c shows the reflected power in a two-tone measurement, similar to Fig. 3a of the main article. Here the main resonance mode shifting downwards at input powers 10^3 aW and higher is less visible. In addition, there is a response shifting to higher frequencies at these powers, similar to what is observed in Ref. 1. As in Fig. 3a, the second resonance mode appearing at lower frequencies is split at sufficiently high drive power. A dashed line indicates where the line-cut at $P_1 = 2,200$ aW in Supplementary Figure 1d is taken. Here we see that we have close-to unity reflection when the drive is weak ($P_1 = 11$ aW) and that the reflection coefficient decreases to ~ 60 % when $P_1 = 2,200$ aW. Again the effects of standing waves in the cabling of the measurement setup are visible. The variations can be seen more clearly in Supplementary Figure 1c than in Supplementary Figure 1a as the z-axis has a different scaling.

SUPPLEMENTARY NOTE 2: POWER CALIBRATION DETAILS

As mentioned in the main article, a power calibration is made as described in Ref. 2. A small difference is that instead of estimating the output power through linear interpolation at the resonance frequency, the resonance frequency is tuned away with magnetic flux during the calibration process. Supplementary Figure 2 presents this calibration measurement. It shows the measured reflected power at the data acquisition (DAQ) system P_m (blue), normalized by the power sent in by the RF generator P_{RF} for the input signal as a function of frequency. Since the resonance frequency is tuned away, the device has unity reflection. The black horizontal line indicates the used calibration line, defining the P_m value for unity reflection $R = 1$ and, for transmission measurements, unity transmission $T = 1$. The measured P_m agrees within ± 20 % of the calibrated value in the range $4.3 - 4.9$ GHz. The reflection coefficient measurement can be used here for the transmission measurement calibration too, since the RF lines of the two ports are identically built.

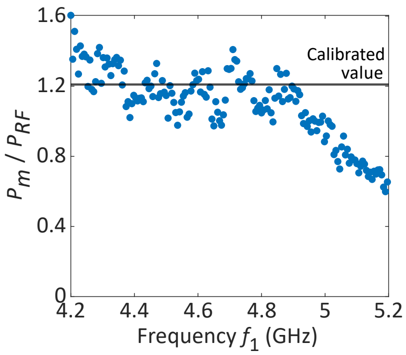

Supplementary Figure 2. Measured reflected power P_m (in blue) at DAQ normalized by the power P_{RF} generated by the RF generator as a function of frequency. The horizontal line at $P_m/P_{RF} = 1.2$ indicates the used calibration value.

[1] T. Yamaji, S. Kagami, A. Yamaguchi, T. Satoh, K. Koshino, H. Goto, Z. R. Lin, Y. Nakamura, and T. Yamamoto, Spectroscopic observation of the crossover from a classical duffing oscillator to a kerr parametric oscillator, Phys. Rev. A **105**, 023519 (2022).

[2] S. Haldar, D. Zenelaj, P. P. Potts, H. Havir, S. Lehmann, K. A. Dick, P. Samuelsson, and V. F. Maisi, Microwave power harvesting using resonator-coupled double quantum dot photodiode, Phys. Rev. B **109**, L081403 (2024).
